# Lightweight and High-Performance Microwave Absorber Based on 2D WS_2_–RGO Heterostructures

**DOI:** 10.1007/s40820-019-0270-4

**Published:** 2019-05-09

**Authors:** Deqing Zhang, Tingting Liu, Junye Cheng, Qi Cao, Guangping Zheng, Shuang Liang, Hao Wang, Mao-Sheng Cao

**Affiliations:** 1 School of Materials Science and Engineering, Qiqihar University, Qiqihar, 161006 People’s Republic of China; 2 Guangdong Provincial Key Laboratory of Micro/Nano Optomechatronics Engineering, College of Mechatronics and Control Engineering, Shenzhen University, Shenzhen, 518060 People’s Republic of China; 3 Department of Chemistry, School of Science, The University of Tokyo, Tokyo, 113-8656 Japan; 4 Department of Mechanical Engineering, School of Engineering, The University of Tokyo, Tokyo, 113-8656 Japan; 5 Department of Mechanical Engineering, Hong Kong Polytechnic University, Hung Hom, Kowloon, Hong Kong People’s Republic of China; 6 School of Materials Science and Engineering, Beijing Institute of Technology, Beijing, 100081 People’s Republic of China

**Keywords:** 2D WS_2_ nanosheets, Reduced graphene oxide, Heterostructure, Microwave absorption

## Abstract

Two-dimensional (2D) nanomaterials are categorized as a new class of microwave absorption (MA) materials owing to their high specific surface area and peculiar electronic properties. In this study, 2D WS_2_–reduced graphene oxide (WS_2_–rGO) heterostructure nanosheets were synthesized via a facile hydrothermal process; moreover, their dielectric and MA properties were reported for the first time. Remarkably, the maximum reflection loss (RL) of the sample–wax composites containing 40 wt% WS_2_–rGO was − 41.5 dB at a thickness of 2.7 mm; furthermore, the bandwidth where RL < − 10 dB can reach up to 13.62 GHz (4.38–18 GHz). Synergistic mechanisms derived from the interfacial dielectric coupling and multiple-interface scattering after hybridization of WS_2_ with rGO were discussed to explain the drastically enhanced microwave absorption performance. The results indicate these lightweight WS_2_–rGO nanosheets to be potential materials for practical electromagnetic wave-absorbing applications.

**Electronic supplementary material:**

The online version of this article (10.1007/s40820-019-0270-4) contains supplementary material, which is available to authorized users.

## Highlights

- WS_2_–rGO nanosheets with ultra-small thicknesses and ultra-lightweight, were successfully prepared by a facile hydrothermal method.
- The WS_2_–rGO isomorphic heterostructures exhibited remarkable microwave absorption properties.

**Electronic supplementary material:**

The online version of this article (10.1007/s40820-019-0270-4) contains supplementary material, which is available to authorized users.

## Graphical abstract

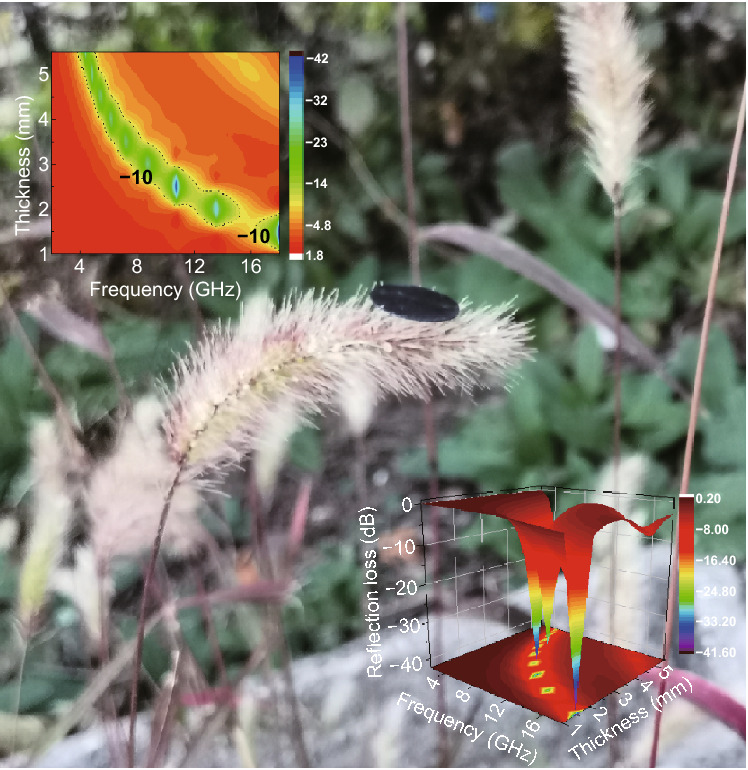

## Introduction

The rapid development of modern industries has highlighted the significance of and the urgent need for developing high-performance electromagnetic wave absorbents with strong absorption, large bandwidth, small thickness, low density, and high temperature/corrosion resistance. Nonetheless, the use of single-component and conventional absorbing materials is not adequate to satisfy the social and industrial requirements [1–7]. Therefore, substantial efforts have been devoted to exploiting novel absorbing materials; these the two-dimensional (2D) materials with ultrathin-layered structures have attracted significant attention owing to their large surface areas and unique electronic properties [8–15]. For example, molybdenum disulfide (MoS_2_), a transition metal dichalcogenide (TMD), has a layered structure similar to that of graphite [16–19]. MoS_2_ nanosheets [20, 21] obtained via the top-down exfoliation method have been reported to exhibit remarkable dielectric properties and absorption performance owing to the dipole polarization at defective sites (*e.g.*, Mo and S vacancies) and ultra-large surface area [22, 23]. In addition to the characteristics of 2D materials [24], such as the quantum and finite-size effects as well as surface and boundary effects, these exhibit high chemical stability and electrical conductivity. The electronic properties of TMDs can be varied from metallic to semiconducting by modulating the crystal structure or layer numbers; this further indicates their substantial potential for application in microwave absorption. However, 2D transition metal sulfides exhibit low electrical conductivity and may not achieve the most suitable impedance matching. It is challenging for single-component dielectric materials to achieve satisfactory microwave absorption performance without being coupled with other dielectric or magnetic components. Thus, introducing the second component into TMD-based absorbents appears significant for further enhancement in their microwave absorption properties [25–27].

Recently, carbon materials [28] including carbon nanotubes (CNTs) [29–32], carbon fibers, and other carbon nanocomposites [33–35] have also been widely investigated for microwave absorption owing to their favorable properties such as low densities and high complex permittivity. Considering this, reduced graphene oxide (rGO) is likely to be an effective complementary material [36]. In a previous work, it was reported that an rGO/CNTs composite with a three-dimensional (3D) nanoarray structure exhibited remarkable microwave absorption performance [37]. Moreover, a composite paper composed of Co_3_O_4_ nanocubes and rGO, when used as microwave absorbent, achieved a maximum reflection loss (RL) of − 31.7 dB at a thickness of 2.5 mm [38]. In addition, a solvothermal-synthesized MoS_2_/rGO heterostructure nanosheet absorber has been reported to exhibit a maximum RL of − 31.57 dB at a thickness of 2.5 mm [39]. Similarly, WS_2_–rGO heterostructure nanosheets could also be synthesized by the solvothermal method [34]; these nanosheets have been used for electrocatalytic hydrogen evolution [40–43]. However, the dielectric loss and microwave absorption properties of WS_2_–rGO systems have not been studied.

In this work, WS_2_–rGO heterostructure nanosheets are synthesized and are used as microwave absorbers for the first time. The coupling of WS_2_ with rGO, which results in the formation of WS_2_–rGO heterostructure nanosheets, is demonstrated to be a facile approach for dramatically improving the microwave absorption performance of WS_2_; it yields a maximum RL of − 41.5 dB at 9.5 GHz with a thickness of 2.7 mm, and a large effective bandwidth of 3.5 GHz with a thickness of 1.7 mm. Based on the systematic structural characterization and electromagnetic measurements, the mechanisms responsible for the superior performances of those heterostructures are proposed. The thin and lightweight WS_2_–rGO heterostructure nanosheet composites in conjunction with their facile synthesis route indicate the numerous potential for developing novel lightweight and high-performance microwave absorption devices.

## Experimental Details

### Materials

All the reagents were of analytical grade and used without further purification. Tungsten hexachloride (WCl_6_) was obtained from Aladdin Industrial Corporation. Thioacetamide (CH_3_CSNH_2_) was supplied by Tianjin Guangfu Fine Chemical Research Institute. Graphene oxide (GO, concentration: 2 mg/mL) was supplied by Xianfeng Nano. Hydrazine hydrate (NH_2_·NH_2_·H_2_O, content ≥ 50%) was obtained from Shenyang Xinxi Reagent Plant. Anhydrous ethanol was obtained from Tianjin Tianli Chemical Reagent Co., Ltd. Deionized water was obtained from Qiqihar City Tianyiyuan Water Plant.

### Preparation of WS_2_ Nanosheets

Tungsten chloride (WCl_6_) (2.0 g) and thioacetamide (4.0 g) were added to 75 mL of deionized (DI) water. The mixture solution was stirred for 30 min and then transferred to a polyphenylene (PPL) autoclave (100 mL), which was heated to 210 °C and maintained at this temperature for 20 h. Subsequently, it was cooled down to room temperature naturally. The black precipitate was extracted from the solution by centrifugation, washed several times with DI water and ethanol, and finally dried in vacuum at 60 °C for 12 h. The sample was denoted as pristine WS_2_ nanosheets and used for comparison with the WS_2_–rGO heterostructure nanosheets.

### Preparation of Reduced Graphene Oxide (rGO)

To obtain rGO, a graphene oxide (GO) dispersion was sonicated for 2 h. Then, hydrazine hydrate was added dropwise to the GO dispersion at room temperature. The reduction was performed at 100 °C for 1 h. The weight ratio of 9:7 for hydrazine hydrate and GO was applied to prepare the sample. The resulting black precipitate was filtered using a filtering paper and washed with anhydrous ethanol and DI water till a neutral pH was attained. Finally, the filtrated precipitate was dried at room temperature for 24 h. The sample was denoted as rGO and used for comparison with the WS_2_–rGO heterostructure nanosheets.

### Preparation of WS_2_–rGO Heterostructure Nanosheets

The WS_2_–rGO heterostructure nanosheets were prepared by a one-pot hydrothermal method. Briefly, 1.5 g of WCl_6_, 3.5 g of thioacetamide, and 30 mL of GO dispersion (2 mg mL^−1^) were mixed; then, DI water was added, achieving a total volume of 75 mL. The mixture solution was stirred for 30 min and then transferred to a 100-mL PPL autoclave, which was heated to 210 °C and maintained at this temperature for 20 h. Subsequently, it was naturally cooled down to room temperature. The resulting black precipitate was recovered from the solution by centrifugation, washed several times with DI water and ethanol, and finally dried in vacuum at 60 °C for 12 h. The sample was denoted as WS_2_–rGO heterostructure nanosheets.

### Characterizations

The morphology and crystal structure of the as-synthesized products were characterized by transmission electron microscopy (TEM, Hitachi, H-7650) and high-resolution TEM (HRTEM, FEI, Tecnai F30). X-ray photoelectron spectroscopy (XPS) analysis was carried out on an X-ray photoelectron spectrometer (ESCALAB250Xi, Thermofisher Co). The X-ray diffraction (XRD) patterns were recorded using a German Bruker-AXS D8 X-ray diffractometer with Cu-K_*α*_ radiation (*λ* = 0.1541 nm). Raman spectroscopy measurements were conducted on a LabRAM HA Evolution system. The defects of the material were analyzed by electron paramagnetic resonance (EPR, Bruker, ER200-SRC-10/12) and fluorescence spectrometer (PL, Hitachi, F7000). The absorption properties of the as-synthesized product were characterized using an ultraviolet spectrophotometer (UV–Vis, PE Company, Lambda 750). The specific surface area and pore size of the WS_2_–rGO heterostructure nanosheets were analyzed using a specific surface area and void analyzer (BET, Quantachrome, NOVA 2000E). The thickness of the as-synthesized product was analyzed by atomic force microscopy (AFM, Bruker, Dimension Edge). The electromagnetic absorption characteristics of the sample were determined by a coaxial method using a vector network analyzer (VNA, MS4644A Anritsu) in the frequency range of 2–18 GHz. Typically, a mixture containing 40 wt% of the as-prepared WS_2_–rGO heterostructure nanosheets and 60 wt% wax was prepared and compressed using a mold to form a ring with an inner diameter of 3 mm and an outer diameter of 7 mm. A 2-mm-thick coaxial center ring was used to evaluate the electromagnetic wave absorption characteristics.

## Results and Discussion

### Morphological, Structural, and Phase Characterization

**Fig. 1:**
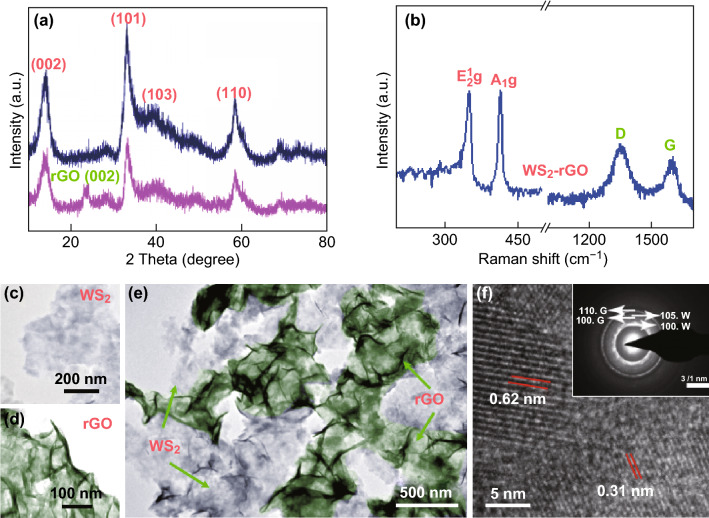
**a** XRD patterns of WS_2_ and WS_2_–rGO. **b** Raman spectrum of WS_2_–rGO. TEM images of **c** WS_2_, **d** rGO, **e** WS_2_–rGO. **f** HRTEM image of WS_2_–rGO heterostructure nanosheets and the corresponding SAED patterns (inset)

In order to identify the phase and crystal structure of the WS_2_–rGO heterostructure nanosheets, characterization based on XRD and Raman spectrum was performed. The results are shown in Fig. 1. In Fig. 1a, several apparent diffraction peaks at 2*θ* = 14.2°, 33.6°, 39.5°, and 59.0° could be identified; these correspond to the crystal planes of (002), (101), (103), and (110), respectively, of WS_2_. These peak positions are reasonably consistent with those of the WS_2_ standard card (PDF No. 08-0237), demonstrating the phase purity of WS_2_ in the as-synthesized product. In the XRD patterns of the WS_2_–rGO heterostructure nanosheets, the additional peak corresponding to the (002) planes of rGO appears at 2*θ* = 24.1° as compared to that of pristine WS_2_. Moreover, it is observed in Fig. S1A that the characteristic peak of pristine rGO also appears at 2*θ* = 24.1°, indicating that the rGO was doped into WS_2_ successfully. It is apparent that the positions of the main diffraction peaks of WS_2_ do not change, except that the intensity of the peak at 2*θ* = 14.2° corresponding to the (002) planes of WS_2_ decreases evidently. The XRD results indicate that the addition of rGO could have significantly suppressed the stacking of the (002) planes of WS_2_ during the solvothermal process; this could have consequently promoted the formation of ultrathin 2D layers of WS_2_ [44, 45]. Meanwhile, owing to the introduction of rGO, the intensity of each diffraction peak of the composite material is weakened. It could have been caused by the introduction of rGO, resulting in the increases in defect density and hence the deterioration of the crystal quality of WS_2_.

The successful formation of WS_2_–rGO compounds was also revealed by Raman spectroscopy. The Raman spectrum of the WS_2_–rGO heterostructure nanosheets (shown in Fig. 1b) displays two characteristic peaks at 1356 and 1598 cm^**−**1^; these are compared with that of the pristine rGO (Fig. S1B). The ratio of the intensities of the D- and G- bands (*I*_D_/*I*_G_) is commonly used to estimate the degree of structural defects in reduced graphene oxides; in this work, it is calculated to be 1.05, which is significantly higher than that of rGO (*I*_D_/*I*_G_ = 0.94). The results indicate the presence of a large number of dangling bonds and defects in the rGO after complexation with WS_2_. Moreover, distinct *E*_2g_ and *A*_1g_ peaks for WS_2_ are observed at 355 and 421 cm^**−**1^. The blueshift in the A_1_g mode can be attributed to the increase in the van der Waals interactions between the layers. Meanwhile, the anomalous behavior of the *E*_2g_ mode can be attributed to the strong dielectric screening of the long range Coulombic interactions between the effective charges in the samples. Thus, the XRD and Raman results adequately demonstrate that rGO was successfully incorporated into the 2D WS_2_ nanosheets during the one-pot solvothermal reaction [26]. It was also verified by the infrared spectroscopy (Fig. S2) that GO was effectively reduced to rGO and that WS_2_ and rGO were effectively combined to form the heterostructures.

A typical TEM image of the as-prepared WS_2_–rGO heterostructure nanosheets is presented in Fig. 1e. It is observed that the loading of rGO on WS_2_ was uniform as compared to that of pure WS_2_ (Fig. 1c) or rGO (Fig. 1d). In those heterostructure nanosheets, rGO retained the wrinkled structure even when it was grown in situ on the surface of WS_2_. Meanwhile, WS_2_ exhibited a layered, broken cotton-like structure. Notably, this growth process endowed WS_2_ nanosheets the maximum contact area with rGO, resulting in a high electrical conductivity of the heterostructure. The conductivity of the heterostructure nanosheet was measured with a four-probe station and observed to be 10 s cm^−1^; meanwhile, that of pure WS_2_ was determined to be 3.33 s cm^−1^, as listed in Table S1. This indicates that the coupling between WS_2_ and rGO could increase the conductivity of the WS_2_ nanosheets. In addition, the presence of graphene suppressed the stacking of the (002) planes of WS_2_, enabling the WS_2_–rGO compounds to grow into relatively thin sheets. Because the interlayer spacing was different across the layered materials, it could be employed to identify the different phases in the heterostructures. As revealed by the HRTEM image and the corresponding selected-area electron diffraction (SAED) patterns (inset of Fig. 1f), the interlayer spacing of approximately 0.62 and 0.31 nm could be indexed to adjacent WS_2_ and rGO layers, respectively. Meanwhile, the diffraction rings in the SAED patterns could also be indexed to the (100) and (105) planes of WS_2_ as well as the (100) and (110) planes of rGO; this further verified the coexistence of WS_2_ and rGO in the heterostructure nanosheets.

**Fig. 2:**
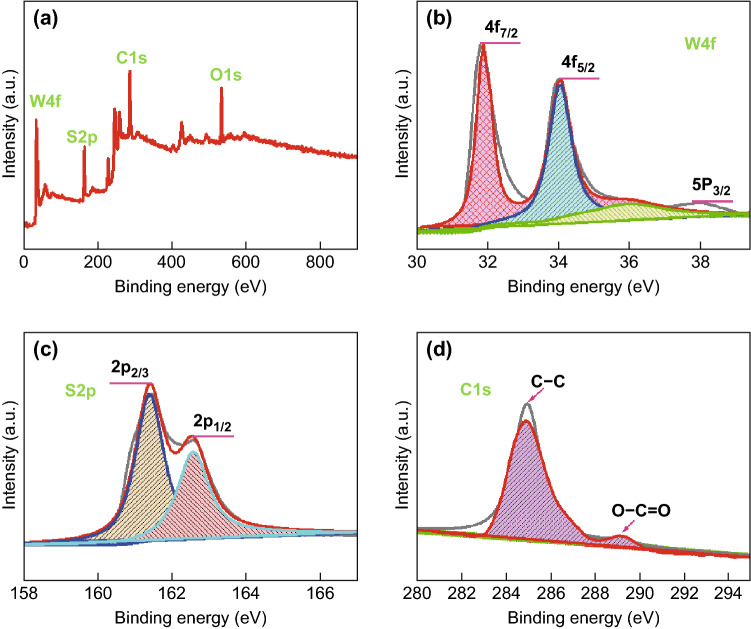
Typical XPS spectra of as-synthesized WS_2_–rGO heterostructure nanosheets: **a** XPS wide scan (survey), high-resolution narrow scan on **b** W, **c** S, and **d** C regions in spectra of WS_2_–rGO

In order to further verify the phases, chemical states, and bonding properties of the WS_2_–rGO heterostructure nanosheets, XPS analysis was performed (Fig. 2). As anticipated, signals of only W, S, C, and O were detected in the survey spectrum (Fig. 2a). The atomic percentages of W, S, C, and O deduced from the survey spectrum were 12.57%, 24.23%, 53.53%, and 9.67%, respectively. The W:S ratio is very close to 1:2, indicating that the stoichiometric WS_2_ was obtained (Fig. S3). The low content of O indicates that GO was effectively reduced to rGO during the solvothermal synthesis process. In addition, as shown in Fig. 2d, the signals resulting from O-bonded C were significantly suppressed in the recorded C 1*s* spectrum of the heterostructure nanosheets. The results further verified the low O-content in the heterostructures. Furthermore, it was observed that the binding energies of W 4*f*_7/2_ and W 4*f*_5/2_ were 31.80 and 34.10 eV, respectively (Fig. 2b). Meanwhile, a weak peak of W 5*p*_3/2_ could be observed at a higher binding energy of approximately 38.00 eV (Fig. 2b). The binding energies of S 2*p*_3/2_ and S 2*p*_1/2_ were measured to be 161.20 and 162.90 eV, respectively (Fig. 2c). All the binding energy values are in good agreement with the standard values; they further demonstrate the presence of W and S in the chemical states of W^4+^ and S^2**−**^, respectively [43].

### Dielectric Properties

**Fig. 3:**
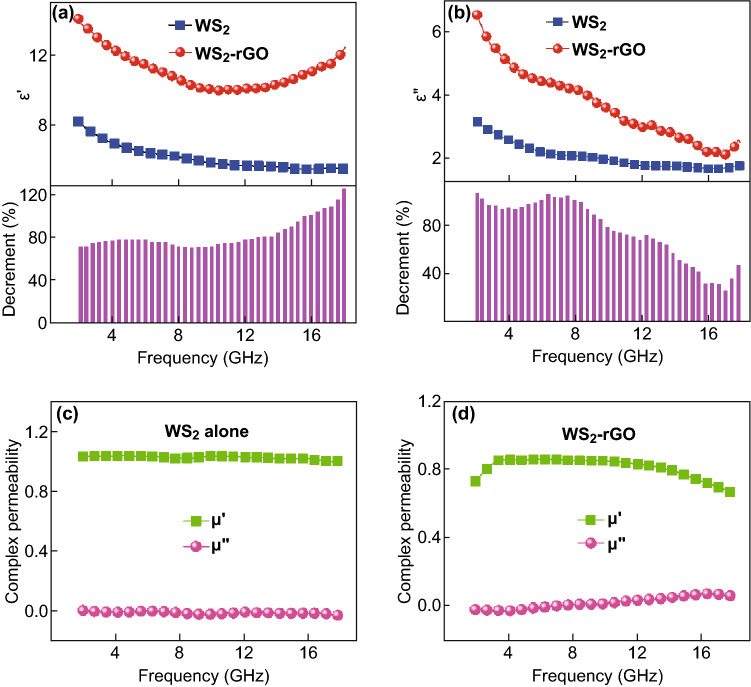
**a** *ε*′-curves, and **b** *ε*″-curves of the as-synthesized WS_2_ and WS_2_–rGO. Complex permeability profiles of **c** pure WS_2_, and **d** WS_2_–rGO

To investigate the dielectric properties of the as-prepared WS_2_ and WS_2_–rGO, dielectric frequency spectra were measured in the frequency range of 2–18 GHz by the coaxial method on an Anritsu MS4644A vector network analyzer. The complex permittivities of WS_2_ and WS_2_–rGO are shown in Fig. 3. It is evident that after hybridization of WS_2_ with rGO, the real permittivity (*ε*′) became higher than that of WS_2_. Moreover, the imaginary permittivity (*ε*″) decreased with increasing frequency. In general, the dielectric loss could be expressed using Debye theory [46], and *ε*′ and *ε*″ can be described as follows:

1 $$\varepsilon^{\prime } = \varepsilon_{\infty } + \frac{{\varepsilon_{s} - \varepsilon_{\infty } }}{{1 + \omega^{2} \tau^{2} }}$$

2 $$\varepsilon^{\prime \prime } = \frac{{\varepsilon_{s} - \varepsilon_{\infty } }}{{1 + \omega^{2} \tau^{2} }}\omega \tau + \frac{\sigma }{{\omega \varepsilon_{0} }}$$

where *ω* is the angular frequency, *τ* is the polarization relaxation time, *ε*_s_ is the static permittivity, and *ε*_∞_ is the relative dielectric permittivity at the high frequency limit.

According to Eq. (1), the decrease in *ε*′ was owing to the increase in *ω* in the testing frequency range [47]. This phenomenon can be related with the polarization relaxation at the low-frequency range. Particularly, when rGO was not added, *ε*′ of WS_2_ was approximately 8.0; moreover, *ε*′ of rGO was approximately 7.0 (Fig. S1C). After the addition of rGO, *ε*′ apparently improved to approximately 14.0. Meanwhile, *ε*″ also increased from approximately 3.0 to 7.0, and the corresponding decremental attained 70–119% and 30–101%, respectively. The results indicated that the addition of rGO and the consequent heterostructurization in the WS_2_–rGO nanosheets significantly improved the dielectric properties of the WS_2_ nanosheets **(**Fig. 3a, b); this could be explained by the interfacial polarization in the heterostructures. The interfacial polarization depends on the difference in the conductivity between two components in the heterostructures, which could enhance the dipole polarization [48]. The increases in *ε*′ and *ε*″ with the increasing loading of rGO could be interpreted rationally according to the effective medium theory. Equation (2) illustrates that *ε*″ is determined by the polarization and electrical conductivity (*σ*); moreover, the relaxations in the frequency range of 2–18 GHz were caused by the polarization of defect, as reported previously [49]. The addition of rGO introduced clustered defects and residual bonds during its heterostructurization with WS_2_; this is likely to have increased the attenuation of electromagnetic waves. Furthermore, because rGO exhibits higher electron mobility, the heterostructures could exhibit a higher hopping conductivity; this in turn caused strong polarization and the loss of conductance in electromagnetic waves. In addition, the coupling between rGO and WS_2_ provided more conductive paths for the heterostructures, which substantially increased the dielectric loss as well.

**Fig. 4:**
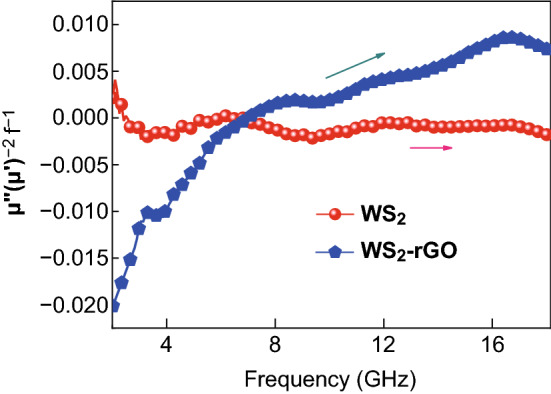
*μ*″(*μ*′)^−2^*f*^−1^ values of WS_2_ and WS_2_–rGO at various frequencies

The magnetic permeability profiles of WS_2_ and WS_2_–rGO are shown in Fig. 3c, d. It is evident that as compared to the real and imaginary permittivity profiles shown in Fig. 3a, b, the magnetic permeability exhibited negligible change prior to and after the heterostructurization of WS_2_ and rGO; this indicates that the addition of rGO had no apparent influence on the magnetic properties of WS_2_. Owing to the absence of magnetism in WS_2_ and WS_2_–rGO, *u*′ and *u*″ were close to one and zero, respectively. *u*′ and *u*″ were close to one and zero for the pristine rGO as well, as shown in Fig. S1D. In general, the eddy current loss played an important role in the magnetic loss at 2.0–18.0 GHz. *u*′ and *u*″ are observed to be distinguished from the curve of the standard spectrum, indicating that the eddy currents were unavoidable. When the magnetic loss is caused only by the eddy current loss, *μ*″(*μ*′)^−2^*f*^−1^ does not change with the frequency. The calculated eddy current loss is shown in Fig. 4. It is observed that *μ*″(*μ*′)^−2^*f*^−1^ varies with frequency, indicating that the WS_2_–rGO heterostructure nanosheets attenuated electromagnetic waves by eddy current loss and relaxation. Meanwhile, Fig. 3d shows that *μ*′ and *μ*″ were very low for the WS_2_–rGO heterostructure nanosheets. The results thus indicate that the electromagnetic wave absorption property of the WS_2_–rGO heterostructure nanosheets mainly resulted from the dielectric loss rather than the magnetic loss.

### Microwave Absorption of WS_2_–rGO

In general, dielectric relaxation could enhance the microwave absorption properties of materials. The reflection loss (RL) of WS_2_–rGO heterostructure nanosheets was evaluated via Eq. (3) [39]:

3 $${\text{RL}} = 20\log \frac{{\left| {Z_{\text{in}} - Z_{0} } \right|}}{{Z_{\text{in}} + Z_{0} }}$$

where the normalized input impedance (*Z*_in_) is expressed as Eq. (4):

4 $$Z_{\text{in}} = \sqrt {\frac{{\mu_{\text{r}} }}{{\varepsilon_{\text{r}} }}} \tanh \left[ {j\frac{2\pi }{c}\sqrt {\mu_{\text{r}} \varepsilon_{\text{r}} } fd} \right]$$

where *Z*_0_ is the impedance of air; *Z*_in_ is the input impedance of the sample; *c* is the velocity of light; ƒ is the electromagnetic wave frequency; *d* is the thickness of the absorbent; and *ε*_r_ and *μ*_r_ are the complex permittivity and permeability of the composite medium, respectively.

**Fig. 5:**
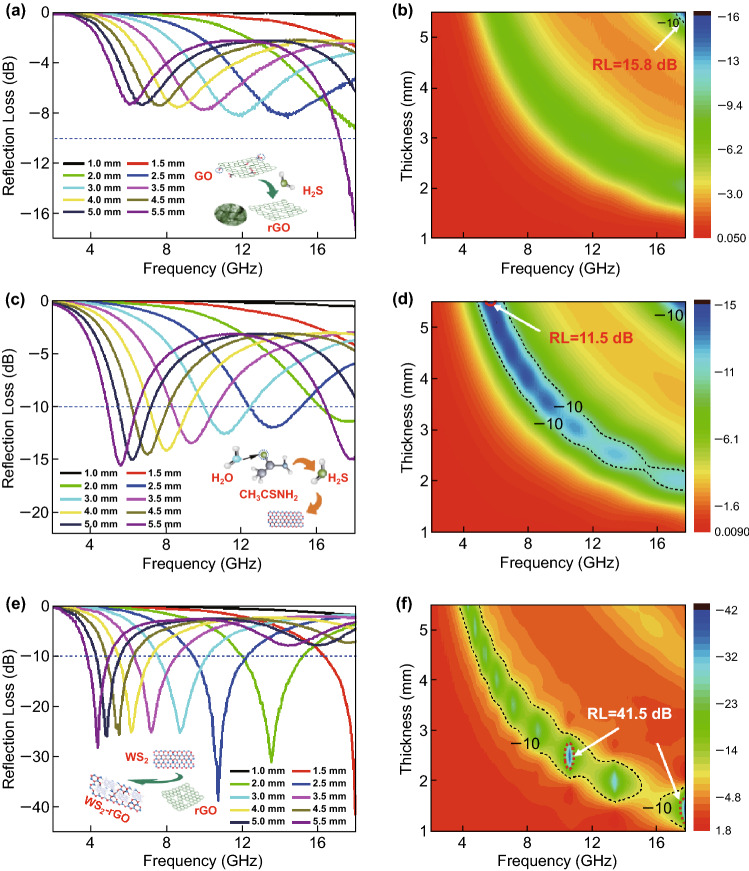
**a**, **c**, **e** Reflection loss profiles and **b**, **d**, **f** corresponding 2D maps of pure rGO (**a**, **b**), pure WS_2_ nanosheets (**c**, **d**), and WS_2_–rGO heterostructure nanosheet powder (**e**, **f**). The illustrations in panels **a**, **c**, and **e** represent different preparation processes for pure rGO, pure WS_2_, and the WS_2_–rGO heterostructure nanosheets, respectively

Figure 5 presents the RL curves at different thicknesses for the sample–wax composites containing 40 wt% rGO (Fig. 5a, b), 40 wt% WS_2_ (Fig. 5c, d), and 40 wt% WS_2_–rGO heterostructure nanosheets (Fig. 5e, f). The microwave absorption performance of rGO was low, considering that it could exceed the effective absorption of − 10 dB only at a large thickness of 5.5 mm at approximately 18 GHz, with a narrow effective bandwidth of 0.85 GHz (17.15–18 GHz) and maximum RL of − 15.8 dB. This is because a remarkable microwave absorbent should exhibit effective electromagnetic attenuation as well as impedance matching characteristics. Although rGO exhibits a high dielectric constant and reasonable electrical conductivity, if its impedance does not match with that of the incident wave, the wave is reflected to the surface of the absorbent, resulting in a weak microwave absorption performance. Therefore, it is very important for the electromagnetic wave-absorbing material to exhibit a suitable impedance matching. Similarly, as shown in Fig. 5c, d, the WS_2_ nanosheets also exhibited weak absorption, with the effective absorption bandwidth of approximately 13 GHz (5–18 GHz) and maximum RL of − 15.5 dB at a large thickness of 5.5 mm. In contrast, as shown in Fig. 5e, f, the microwave absorption properties of the WS_2_–rGO heterostructure nanosheets are remarkable, with significantly stronger RL than that of the absorbents made only of rGO or WS_2_ nanosheets. The maximum RL is − 25 dB at a thickness of 3.0 mm, and the maximum RL even attains − 41.5 dB at a thickness of 1.5 mm. The effective bandwidth is 13.62 GHz (4.38–18 GHz). As listed in Table S2, we compared WS_2_–rGO with other rGO-based and MoS_2_-based MA materials reported in the literature. It is observed that WS_2_–rGO hybrids exhibit remarkable RL values and a broadened bandwidth, indicating the potential applications of WS_2_–rGO hybrids.

**Fig. 6:**
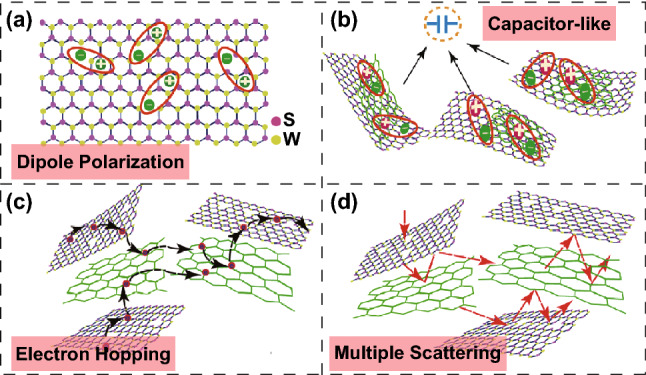
Synergistic mechanisms responsible for the superior microwave absorption properties of the proposed WS_2_–rGO heterostructure nanosheet absorbent: **a** dipole polarization. **b** Interfacial polarization. **c** Electron hopping. **d** Multiple scattering of incident microwave

The dramatic enhancement in the microwave absorption properties could have been caused by the hybridization of rGO with WS_2_, which could regulate the complex permittivity of WS_2_ nanosheets. Meanwhile, there were a large number of defects in the WS_2_ and rGO layers; these were W vacancies and S vacancies in WS_2_ and a few oxygen-containing functional groups in rGO. These resulted in the generation of a large number of dipoles. As illustrated in Fig. 6a, dipole polarization occurred subsequently; it played an important role in microwave attenuation. In addition, owing to the difference in the conductivity of the surfaces of rGO and WS_2_, the local charge accumulation and rearrangement could have resulted in interfacial polarization [50–54] under an alternating electromagnetic field. The interfacial polarization sites (Fig. 6b) could be considered as a type of capacitor-like structure and hence could effectively absorb electromagnetic waves. In addition, as shown in Fig. 6c, electrons could absorb the energy of incident microwave and jump among those heterostructure nanosheets, resulting in eddy current losses for microwave attenuation. Moreover, both WS_2_ and rGO nanosheets exhibited high specific surface area and plenty of interlayer voids owing to their ultrathin 2D structures; this enabled the formation of a favorable multiple-scattering and conductive network (Fig. 6d). Large-scale SEM images (Fig. S4) further reveal the 3D interconnected network structure formed by the WS_2_–rGO heterostructure nanosheets at micrometer scales; this was also favorable toward enhancing microwave dissipation via the multiple scattering effect. The above-mentioned results demonstrate that the introduction of rGO could significantly improve the wave absorption performance as compared with the pristine tungsten disulfide or rGO. This could be attributed to the differences in impedance matching and attenuation constants, which are two critical parameters that affect the value of RL [55, 56]. In order to achieve zero reflection on the surface of the sample, the characteristic impedance of the sample should be equal to or close to that of the free space, which generally depends on a function between the complex permissibility and complex permeability. A delta-function method was used to validate the degree of impedance matching by Eq. (5) [57–59]:

5 $$\left| \Delta \right| = \left| {\sinh^{2} (Kfd) - M} \right|$$

where the values of *K* and *M* could be calculated using the complex permittivity and permeability as Eqs. (6) and (7):

6 $$K = \frac{{4\pi \sqrt {\mu^{\prime } \varepsilon^{\prime } } }}{{c\cos \delta_{\text{e}} \cos \delta_{\text{m}} }}\sin \frac{{\delta_{\text{e}} + \delta_{\text{m}} }}{2}$$

7 $$M = \frac{{4\mu^{\prime } \cos \delta_{\text{e}} \varepsilon^{\prime } \cos \delta_{\text{m}} }}{{(\mu^{\prime } \cos \delta_{\text{e}} - \varepsilon^{\prime } \cos \delta_{\text{m}} )^{2} + \left[ {\tan \left( {\frac{{\delta_{\text{m}} }}{2} - \frac{{\delta_{\text{e}} }}{2}} \right)} \right]^{2} (\mu^{\prime } \cos \delta_{\text{e}} + \varepsilon^{\prime } \cos \delta_{\text{m}} )^{2} }}$$

**Fig. 7:**
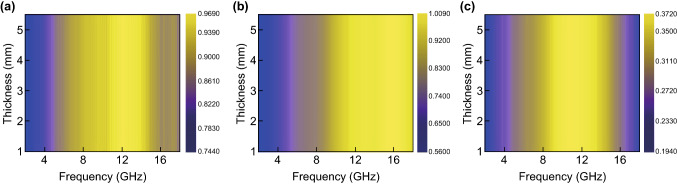
Calculated delta value maps of **a** rGO, **b** WS_2_, and **c** WS_2_–rGO

Generally, when a smaller |*Δ*| is obtained, there is a better impedance match between the complex permittivity and complex permeability [60, 61]. The calculated delta value plots for WS_2_, rGO, and the WS_2_–rGO heterostructure nanosheets at 2–18 GHz are shown in Fig. 7. It is observed that the value for the WS_2_–rGO heterostructure nanosheets is close to zero, indicating a higher impedance matching performance as compared to that of pristine WS_2_ or rGO. Meanwhile, the matching impedance values with the comparison rGO > WS_2_ > WS_2_–rGO are presented in Fig. 7. The results are consistent with the RL results shown in Fig. 5, indicating that the WS_2_–rGO heterostructure nanosheets exhibit advantages in impedance matching.

Figure 5 shows that the RL of pristine rGO or WS_2_ is relatively weak when the thickness is small; meanwhile, the RL of WS_2_–rGO heterostructure nanosheets increased with decreasing thickness. Moreover, the coupling between WS_2_ and rGO also resulted in a shift of the effective absorption band to the low-frequency region. These unique properties enabled the WS_2_–rGO heterostructure nanosheets to achieve the unique features of strong absorption, small thickness, and lightweight. The AFM image further characterizes the thickness of the heterostructure, as shown in Fig. S5 of the supplementary information. Compared to that of WS_2_ nanosheets, the thickness of the heterostructure increased, although the overall thickness was approximately 50 nm. This establishes that the WS_2_–rGO heterostructure nanosheets prepared were particularly thin material. Figure S6 displays a piece of sample made from the as-prepared WS_2_–rGO heterostructure nanosheets; it is placed on a blade of *Setaria viridis*. The fluff of *Setaria viridis* did not exhibit apparent bending; moreover, the sample did not fall. This verified the remarkable lightweight features of the WS_2_–rGO heterostructure nanosheet used as microwave absorbent.

In order to explain the absorption properties of and defect formation in the WS_2_–rGO heterostructure nanosheets, electron paramagnetic resonance (EPR) and photoluminescence (PL) measurements were performed (Fig. S7). Figure S7A shows that the PL peaks for WS_2_, rGO, and the WS_2_–rGO heterostructure nanosheets are located at 388.2, 392.1, and 394.6 nm, respectively. The small luminescence intensity of tungsten disulfide indicates the presence of numerous defects [62]. Compared with that of WS_2_ or rGO, the PL peak for the WS_2_–rGO heterostructure nanosheets exhibits a significant blueshift; this is owing to the reduced number of layers of the WS_2_–rGO heterostructure nanosheets after the heterostructurization. The PL peak intensity for the WS_2_–rGO heterostructure nanosheets is higher than that of WS_2_ or rGO; this indicates that the carrier recombination was strong and that the defect concentration could have been increased. Because the defect sites were more likely to become recombination centers, the charge recombination aggravated. Therefore, the PL peak intensity of the heterostructure increased. Figure S7B shows the EPR spectrum of the WS_2_–rGO heterostructure nanosheets at room temperature. The signal appears at the vicinity of the magnetic field of 3513 G (*g* ≈ 1.9). The narrow signal peaks could be related to the presence of S (Vs) vacancies. The large peak intensity established that the S vacancies had a large defect concentration; this again established that the WS_2_–rGO heterostructure nanosheets had a large number of defects, resulting in dipole polarization losses.

In addition, UV–Vis and BET tests were carried out to characterize the porous WS_2_–rGO heterostructure structure and to further demonstrate the microwave absorbing properties. As shown in Fig. S8A, the absorption spectrum of WS_2_–rGO heterostructure nanosheets exhibits a distinct absorption peak in the wavelength range of 190–400.7 nm. This absorption peak exhibits a significant blueshift as compared to the characteristic absorption peak at 920 nm (with a forbidden band width of 1.35 eV) for the tungsten disulfide bulk material; this is apparently caused by the quantum confinement effect. The strong absorption strength of the WS_2_–rGO heterostructure nanosheets at 190–400.7 nm could have been owing to the high specific surface area of these nanosheets; this was demonstrated by the nitrogen adsorption test. The BET surface areas and pore size distribution curves of the WS_2_–rGO heterostructure nanosheets were obtained by nitrogen adsorption–desorption measurements, as shown in Fig. S8B. The isotherm is of type IV according to the Brunauer–Deming–Deming–Teller classification. Furthermore, the shapes of the hysteresis loops in the *P*/*P*_0_ range of 0.4–1.0 are of type H3, which indicate the formation of porous structures in the samples. The pore size distribution curves indicate that the samples exhibited wide pore size distributions (3–30 nm) with a main pore size of 3.409 nm; this verifies the presence of mesopores in the samples. The BET-specific surface areas of the WS_2_–rGO heterostructure nanosheets were 72.916 m^2^ g^−1^. The illustrations in Fig. 5a, c, e show the models of preparation of rGO, WS_2_, and the WS_2_–rGO heterostructure nanosheets, respectively. The unique processing condition produced tungsten disulfide WS_2_–rGO hybrid material with remarkable performance, ultra-lightweight, ultra-small thickness, and high specific surface. This indicates that the WS_2_–rGO heterostructure nanosheets could have been porous and have had a high specific surface area, providing more spaces for multiple reflections and scattering of electromagnetic waves. The porous structure also facilitated the formation of a uniform complicate conductive network, resulting in remarkable electromagnetic wave absorption in the WS_2_–rGO heterostructure nanosheets.

**Fig. 8:**
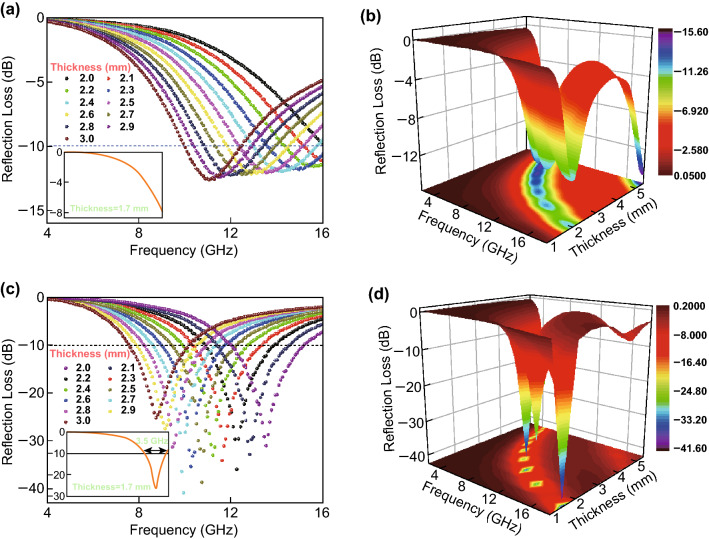
a, **c** Reflection loss profiles and **b**, **d** 3D maps of RL of pristine WS_2_ microwave absorbent at thickness of 2.0–3.0 mm (**a**, **b**) and WS_2_–rGO heterostructure nanosheet absorbent at thickness of 2.0–3.0 mm (**c**, **d**). The insets in panels **a** and **e** show the RL curves of the pristine WS_2_ and the WS_2_–rGO heterostructure nanosheet absorbents at a thickness of 1.7 mm

Figure 8 shows the RL of WS_2_ and the WS_2_–rGO heterostructure nanosheets with thicknesses of 2.0–3.0 mm. The maximum effective bandwidth of the WS_2_–rGO heterostructure nanosheet absorbent is calculated to be 3.5 GHz at a rather small thickness of 1.7 mm, as shown in the inset of Fig. 8c; meanwhile, the effective bandwidth of pristine WS_2_ at this thickness is zero (inset of Fig. 8a). It is further observed in Fig. 8 that with a relatively small thickness of 2.0–3.0 mm, the pristine WS_2_ exhibited low microwave absorption performance as compared to the WS_2_–rGO heterostructure nanosheets. The maximum RL of the WS_2_–rGO nanosheets could have been − 41.5 dB at a thickness of 2.7 mm; this indicates that the heterostructurization of WS_2_ with rGO is an efficient method for improving the absorbing properties (*i.e.,* RL). The aforementioned results thus indicate that WS_2_–rGO heterostructure nanosheets exhibit significant potential for applications as novel high-performance microwave absorbents because of their facile one-pot synthesis route, lightweight, small thickness, low cost, and corrosion and oxidation resistance, in conjunction with their significantly enhanced RL and effective bandwidth.

## Conclusions

In summary, well-defined WS_2_–rGO heterostructure nanosheets were directly synthesized via a facile one-step hydrothermal approach; moreover, their microwave absorption performance was investigated. Significantly enhanced absorption was observed in the WS_2_–rGO heterostructure nanosheet absorbent, as reflected by the high RL and extended effective absorption bandwidth; this could be attributed to the interfacial dielectric coupling at the well-defined WS_2_–rGO interfaces constructed by the introduction of rGO. In particular, it is observed that the absorber made from the WS_2_–rGO heterostructure nanosheets of thickness 2.7 mm achieved the maximum RL of –41.5 dB at 9.5 GHz. More remarkably, a large effective absorption bandwidth of 3.5 GHz could be realized by the heterostructure absorber of thickness approximately 1.7 mm. In addition, the effective bandwidth for the WS_2_–rGO heterostructure nanosheet absorber could be further adjusted from 18 GHz to a low-frequency band by adding rGO. Because of their attractive microwave absorption properties as well as their features of facile synthesis route, small thickness, and lightweight, the WS_2_–rGO heterostructure nanosheets are considered to be potential lightweight and wide-frequency microwave absorption materials.

## Electronic supplementary material

Below is the link to the electronic supplementary material.

Supplementary material 1 (PDF 613 kb)
