# Supplementary material for: Lightweight and High-Performance Microwave Absorber Based on 2D WS2–RGO Heterostructures
Source: Nanomicro Lett. 2019 May 9;11:38. doi: 10.1007/s40820-019-0270-4 (PMC7770953; doi:10.1007/s40820-019-0270-4)
Supplement: Supplementary file 1 — Supplementary material 1 (PDF 613 kb) [file 40820_2019_270_MOESM1_ESM.pdf]

Supporting Information for

Lightweight and High-Performance Microwave Absorber Based on 2D WS_2–RGO Heterostructures

Deqing Zhang[1, 5, †], Tingting Liu[1, †], Junye Cheng[2, *], Qi Cao[3], Guangping Zheng[4], Shuang Liang[1], Hao Wang[2, *], Mao-Sheng Cao[5, *]

[1]School of Materials Science and Engineering, Qiqihar University, Qiqihar 161006, People's Republic of China

[2]Guangdong Provincial Key Laboratory of Micro/Nano Optomechatronics Engineering, College of Mechatronics and Control Engineering, Shenzhen University, Shenzhen 518060, People's Republic of China

[3]Department of Chemistry, School of Science, and Department of Mechanical Engineering, School of Engineering, The University of Tokyo, Tokyo 113-8656, Japan

[4]Department of Mechanical Engineering, Hong Kong Polytechnic University, Hung Hom, Kowloon, Hong Kong, People's Republic of China

[5]School of Materials Science and Engineering, Beijing Institute of Technology, Beijing 100081, People's Republic of China

†These authors contribute equally to this work.

*Corresponding authors. E-mail: whao@szu.edu.cn (Hao Wang); jycheng4-c@my.cityu.edu.hk (Junye Cheng); caomaosheng@bit.edu.cn (Mao-Sheng Cao)

Supplementary Figures and Tables

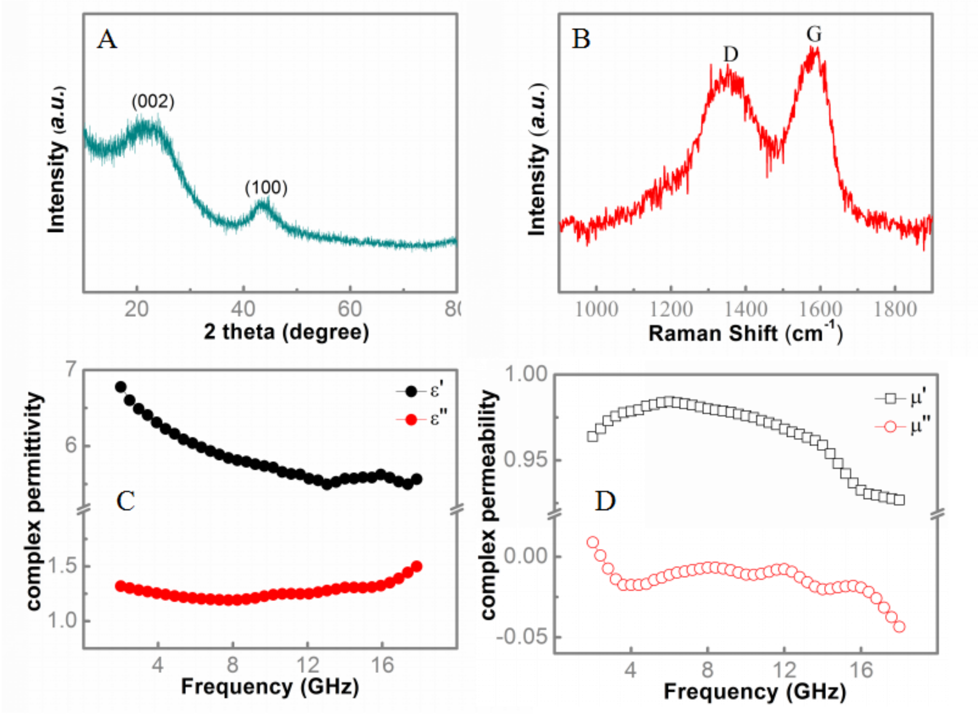

Fig. S1 **A** XRD patterns, **B** Raman spectrum, **C** Complex permitivity profiles, and **D** Complex permeability profiles of rGO

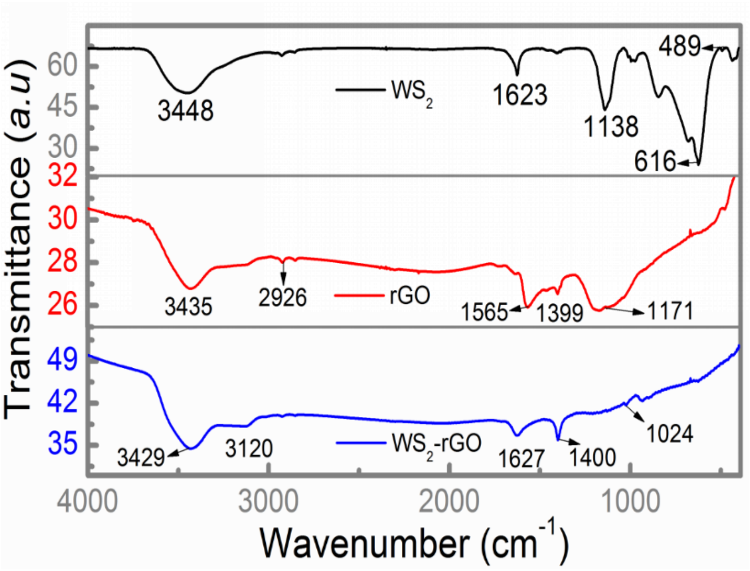

Fig. S2 FI-IR spectra of WS_2, rGO, and WS_2–rGO

The results confirm that, in the hybrid material, GO was well reduced to rGO. For rGO, the stretching vibration peak of -OH appears at 3435 cm^{-1}, the C-H stretching

vibration peak appears at 2926 cm^{-1}, the C-C stretching vibration absorption peak appears at 1565 cm^{-1}, and the O-H stretching vibration peak appears at 1399 cm^{-1}, the C-O stretching vibration peak appears at 1171 cm^{-1}. After the complexation, the peaks of the WS_2–rGO heterostructure nanosheets are shifted, for example, the stretching vibration peak of -OH appears at 3429 cm^{-1}, the stretching vibration peak of C-H appears at 3120 cm^{-1}, the O-H stretching vibration peak appears at 1400 cm^{-1}, and the C-O stretching vibration peak appears at 1024 cm^{-1}. The results suggest that there are interactions among functional groups of rGO and WS_2 crystals and WS_2–rGO heterostructure is well formed. At the same time, there are several characteristic peaks of tungsten disulfide for the WS_2–rGO heterostructure nanosheets. The peak close to at 3448 cm^{-1} is ascribed to the W-S vibration; The peak close to 1623 cm^{-1} is the stretching vibration of -OH resulting from the combination of water.

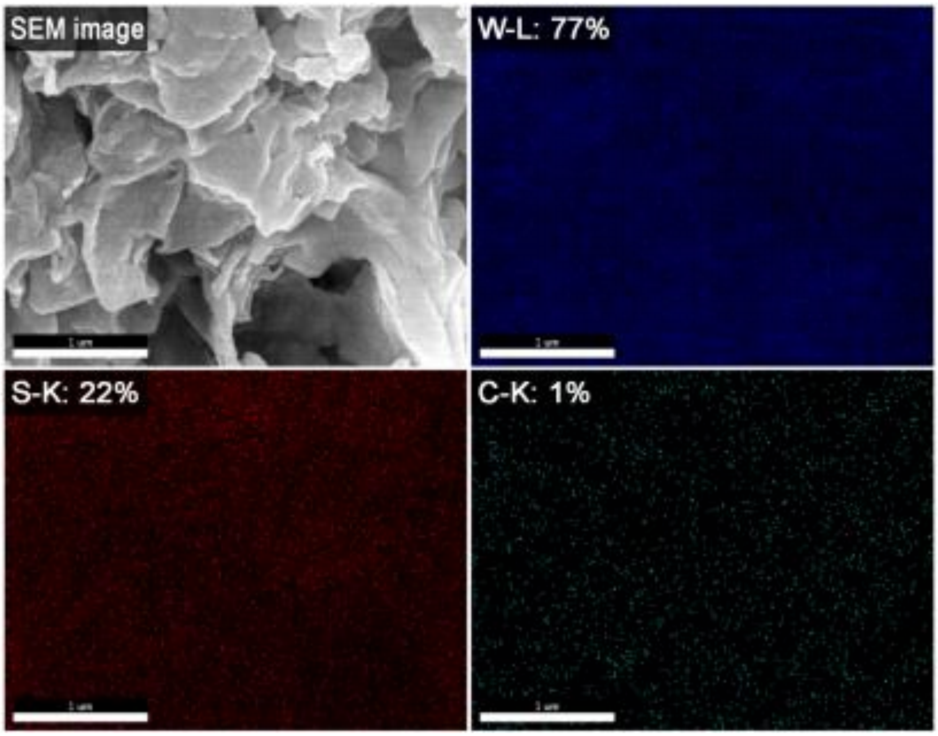

Fig. S3 SEM–EDS elemental mapping profiles of the as-synthesized WS_2–rGO heterostructure nanosheet powders

The results confirm that the elements W and S (in WS_2), and also C (in rGO) distribute uniformly in the as-synthesized WS_2–rGO heterostructure nanosheet powders.

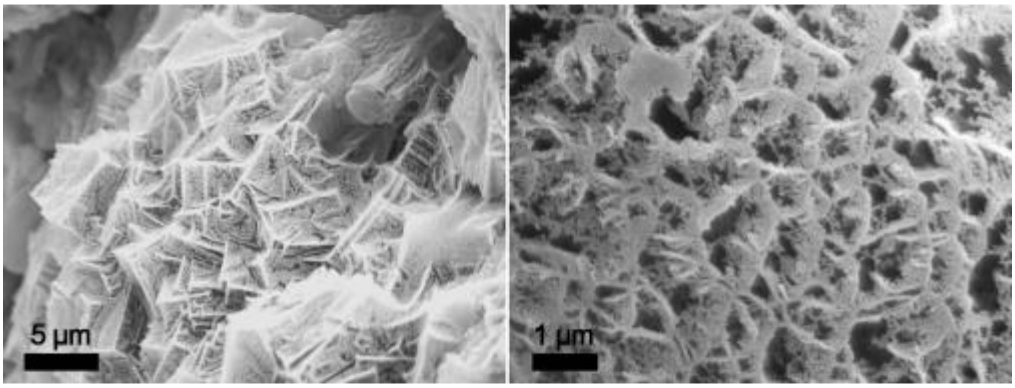

Fig. S4 Large-scale SEM images of the as-synthesized WS_2–rGO heterostructure nanosheet powders at different magnifications

The images reveal the 3D interconnected network structure formed from the as-synthesized WS_2–rGO heterostructure nanosheets at micrometer scales, which might be favorable for enhancing the multiple scattering-induced microwave dissipation, as illustrated in Fig. 8d in the main text.

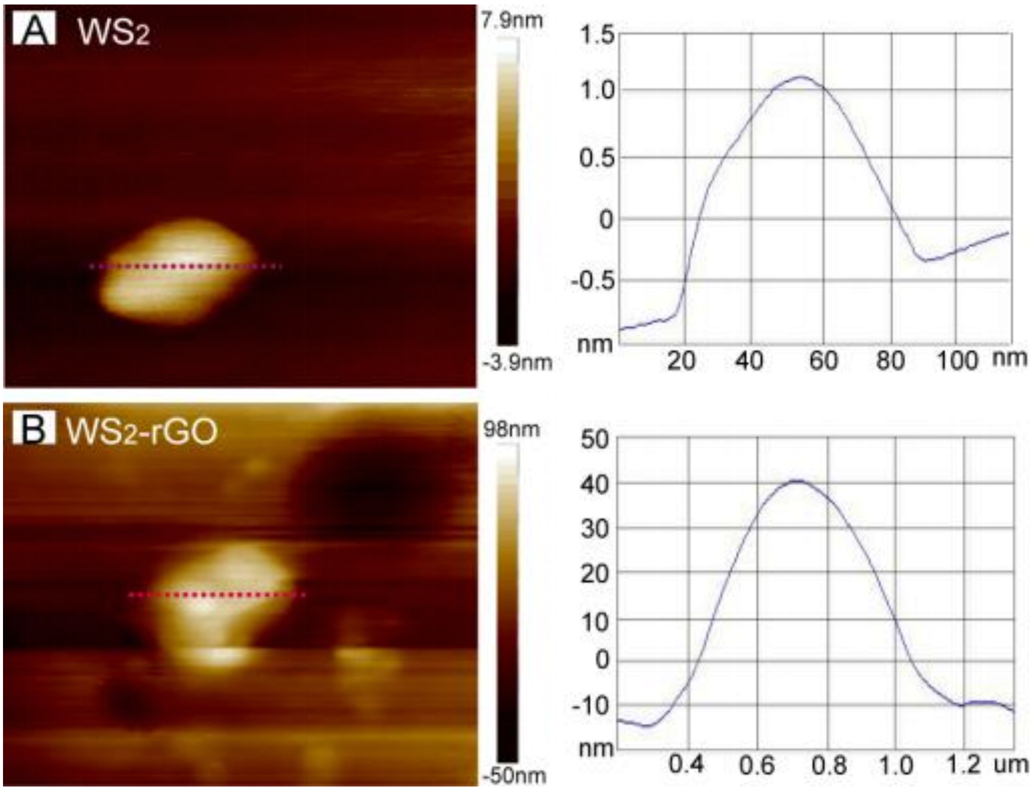

Fig. S5 AFM images of **A** WS_2, **B** WS_2–rGO

Compared to that of pristine tungsten disulfide nanosheets, the thickness of WS_2–rGO is increased, while it is only about 50 nm. The results prove that the WS_2-rGO heterostructure nanosheet prepared is a particularly thin material.

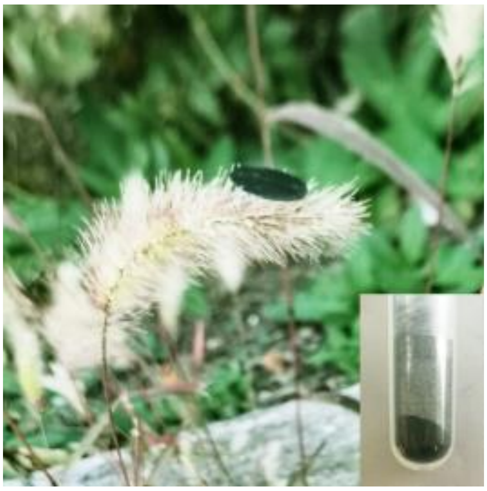

Fig. S6 Optical image of a piece of sample made from the as-synthesized WS_2–rGO heterostructure nanosheets, which is put on a *Setaria viridis*. The inset displays the image of the as-synthesized WS_2–rGO powders

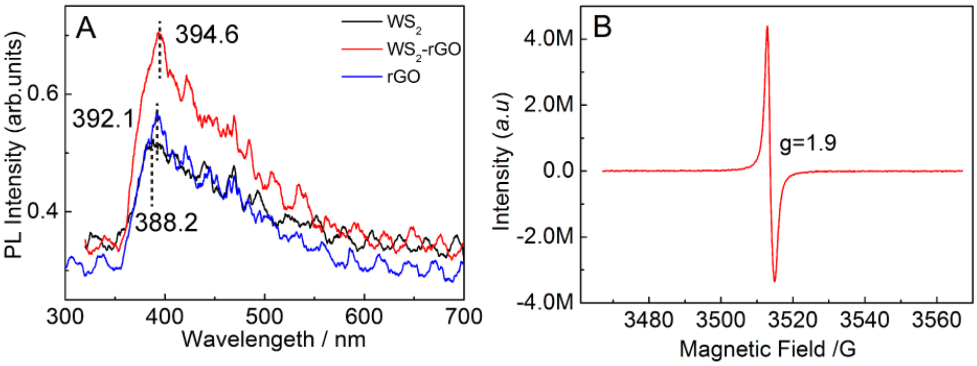

Fig. S7 A Photoluminescence spectra of WS_2, rGO, and the WS_2-rGO heterostructure nanosheets; λ_{exc} = 280 nm; **B** EPR signals of WS_2-rGO heterostructure nanosheets at room temperature

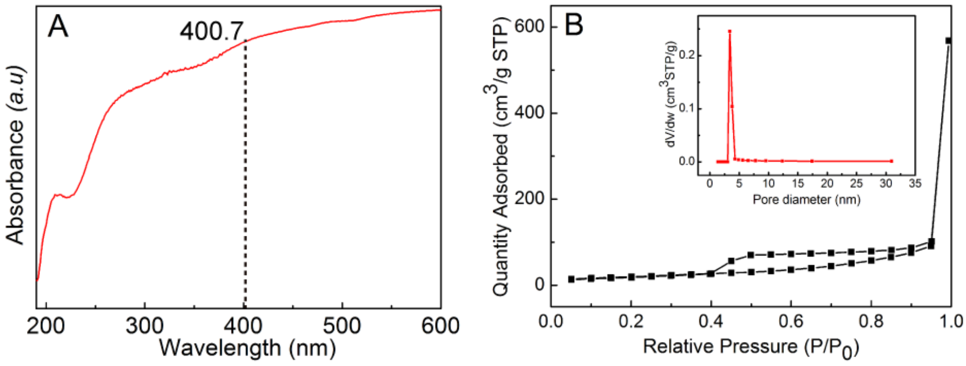

Fig. S8 A UV–Vis spectra of the WS_2-rGO; **B** Nitrogen adsorption-desorption isotherms and the corresponding pore size distribution curves for the WS_2–rGO

Table S1 The measured conductivity of WS_2, rGO, and WS_2–rGO

Sample	WS_2	rGO	WS_2-rGO
σ (s cm^{-1})	3.33	10.86	10

The results demonstrate that WS_2 and rGO have different electrical conductivity, and the electrical conductivity of the WS_2-rGO heterostructure nanosheets is higher than that of the WS_2 nanosheets, which also prove the successful combination of WS_2 and rGO.

Table S2 Microwave Absorption Performance of MA materials

Samples	Matrix	Minimum RL value (dB)	Thickness (mm)	Loading ratio (wt%)	Frequency range (GHz)	Effective bandwidth (GHz)	Refs.
WS_2-rGO	wax	-41.5	1.5	40	4.38-18.0	13.62	This work
RGO	PVDF	-25.6	4.0	3	8.48-12.8	4.32	[S1]
RGO/$CoFe_2O_4$	wax	-38.5	2.0	50	9.2-15.0	5.8	[S2]
MoS_2-NS	wax	-38.42	2.4	60	9.6-13.76	4.16	[S3]
Co_3O_4/RGO	wax	-31.7	2.5	20	5.50-16.00	10.50	[S4]
MoS_2/RGO	wax	-31.57	2.5	10	6.5-18.0	11.5	[S5]
MoS_2/CNT	wax	-46	2.9	50	3.4-13.9	10.5	[S6]

Supplementary References

[S1] X.J. Zhang, G.S. Wang, W.Q. Cao, Y.Z. Wei, M.S. Cao, L. Guo, Enhanced microwave absorption property of reduced graphene oxide (RGO)-$MnFe_2O_4$ nanocomposites and polyvinylidene fluoride. RSC Adv. **6**(10), 7471-7419 (2014). https://doi.org/10.1021/am500862g

[S2] M. Zong, Y. Huang, N. Zhang, H.W. Wu, Facile synthesis of RGO/Fe_3O_4/Ag composite with high microwave absorption capacity. Mater. Lett. **111**(15), 188-191 (2013). https://doi.org/10.1016/j.matlet.2013.08.076

[S3] M.Q. Ning, M.M. Lu, J.B. Li, Z. Chen, Y.K. Dou, C.Z. Wang, F. Rehman, M.S. Cao, H.B. Jin, Two-dimensional nanosheets of MoS_2: a promising material with high dielectric properties and microwave absorption performance. Nanoscale **7**(38), 15734-15740 (2015). https://doi.org/10.1039/c5nr04670j

[S4] X.B. Li, S.W. Yang, J. Sun, P. He, X.P. Pu, G.Q. Ding, Enhanced electromagnetic wave absorption performances of Co_3O_4 nanocube/reduced graphene oxide composite. Synth. Met. **194**, 52-58 (2014). https://doi.org/10.1016/j.synthmet.2014.04.012

[S5] X. Ding, Y. Huang, S. Li, N. Zhang, J. Wang, 3D architecture reduced graphene oxide-MoS_2 composite: preparation and excellent electromagnetic wave absorption performance. Composites Part A **90**, 424-432 (2016). https://doi.org/10.1016/j.compositesa.2016.08.006

[S6] C.Mu, J. Song, B. Wang, C. Zhang, J. Xiang, F. Wen, Z. Liu, Two dimensional materials and one dimensional carbon nanotubes composites for microwave absorption. Nanotechnology **29**(2), 025704 (2017). https://doi.org/10.1088/1361-6528/aa9a2a
